# Ti-V-C-Based Alloy with a FCC Lattice Structure for Hydrogen Storage

**DOI:** 10.3390/molecules24030552

**Published:** 2019-02-02

**Authors:** Bo Li, Liqing He, Jianding Li, Hai-Wen Li, Zhouguang Lu, Huaiyu Shao

**Affiliations:** 1 Joint Key Laboratory of the Ministry of Education, Institute of Applied Physics and Materials Engineering (IAPME), University of Macau, Macau SAR, China; yb77809@umac.mo (B.L.); yb77803@umac.mo (J.L.); 2 Department of Materials Science and Engineering, Southern University of Science and Technology, Shenzhen 518055, China; heli_limao@163.com (L.H.); luzg@sustc.edu.cn (Z.L.); 3 Kyushu University Platform of Inter/Transdisciplinary Energy Research (Q-PIT), Kyushu University, Fukuoka 819-0395, Japan; li.haiwen.305@m.kyushu-u.ac.jp; 4 Department of Physics and Chemistry, Faculty of Science and Technology, University of Macau, Macau SAR, China

**Keywords:** mechanical alloying, metals and alloys, crystal structure, hydrogen absorbing materials

## Abstract

Here we report a Ti_50_V_50_-10 wt.% C alloy with a unique lattice and microstructure for hydrogen storage development. Different from a traditionally synthesized Ti_50_V_50_ alloy prepared by a melting method and having a body-centered cubic (BCC) structure, this Ti_50_V_50_-C alloy synthesized by a mechanical alloying method is with a face-centered cubic (FCC) structure (space group: *Fm-3m* No. 225). The crystalline size is 60 nm. This alloy may directly absorb hydrogen near room temperature without any activation process. Mechanisms of the good kinetics from lattice and microstructure aspects were discussed. Findings reported here may indicate a new possibility in the development of future hydrogen storage materials.

## 1. Introduction

Hydrogen energy is thought to one of most promising energy solutions due to its advantages such as easy conversion from and to electricity, producing only water, zero emission of pollutants and the large energy density of hydrogen (120 MJ/kg for lower heating value) [1,2]. However, hydrogen is in gaseous state at normal conditions and the volumetric energy density is low. One must find a safe, low-cost and highly efficient way to store hydrogen and this is hydrogen storage, which is almost the most critical technology before the universal realization of a hydrogen-based economy [3]. Hydrogen storage in solid-state materials is considered as the most promising technology. Various types of hydrogen storage materials have been studied for these kinds of applications, e.g., chemical storage in the metals and alloys (V, La-Ni, Ti-Fe, Mg/Mg-M, M = metal), M-Al-H, M-N-H and M-B-H systems, or physical adsorption of hydrogen on carbon based materials or metal-organic frameworks (MOFs) [4,5,6]. Ti-V-based alloys with a body-centered cubic (BCC) structure have been intensively investigated as hydrogen storage materials [7,8,9,10,11,12,13,14] because of their characteristics of high hydrogen capacity (4 wt.%, or about 150 kg H/m^3^) and a possibility of working temperatures below 200 °C. The challenges of the Ti-V-based alloys for hydrogen storage lie in their low reversible storage capacity (less than 3 wt.%) and very difficult activation process, when normally a temperature of above 400 °C and a hydrogen pressure above 4 MPa are needed for activation before the Ti-V alloys may absorb hydrogen. 

Mechanical alloying or ball milling techniques are widely adopted in fabrication of hydrogen storage materials in order to synthesize them in convenient conditions, and improve the hydrogen absorption and desorption kinetics [6,15,16,17,18,19,20]. These techniques are well known to be able to form metastable phases, increase surface area and reduce particle/crystallite size. The authors have achieved numerous interesting results in development of metastable nanostructured hydrogen storage materials with unique microstructures through mechanical alloying techniques [21,22,23,24,25]. Nevertheless, one can hardly find any reported result on synthesis of Ti-V-based alloys by mechanical alloying for hydrogen storage research. This is because when Ti and V mixture were directly ball milled using stainless steel balls and vessel, after ball milling for a few to tens of hours, the starting materials will stick on the surface of the vessel wall and the balls. No powder sample can be obtained by this method. Recently, Shao et al. [26] synthesized TiVMn- and TiCrMn-based nanoalloys with a mean particle size around a few to tens of µm, the crystallite size just 10 to 13 nm and the obtained samples showed enhanced hydrogen absorption performance from the high hydrogen pressure differential scanning calorimetry (DSC) results. Here we find that Ti and V mixture could be ball-milled and collected after introduction of small amount of carbon materials. In this work, we report a Ti-V-C-based alloy, not with a BCC structure, but with a face-centered cubic (FCC) structure, which was prepared by carbon added mechanical alloying method. During the cycling of absorption-desorption in hydrogen storage materials, change in the lattice structure and lattice expansion caused by hydrogen atoms makes it difficult to achieve effective and compact packing of the materials. Similar to the phenomenon reported by the authors in Mg-Co based BCC alloys [24,27], this reported Ti-V-C alloy is always with a stable FCC lattice structure before and after hydrogenation and with no big difference in its lattice parameter. This means great potential in reversible and long cycle-life hydrogen storage. The findings in this work may start a new direction for the development of novel hydrogen storage materials with designed lattice structures. 

## 2. Results and Discussion

**Figure 1:**
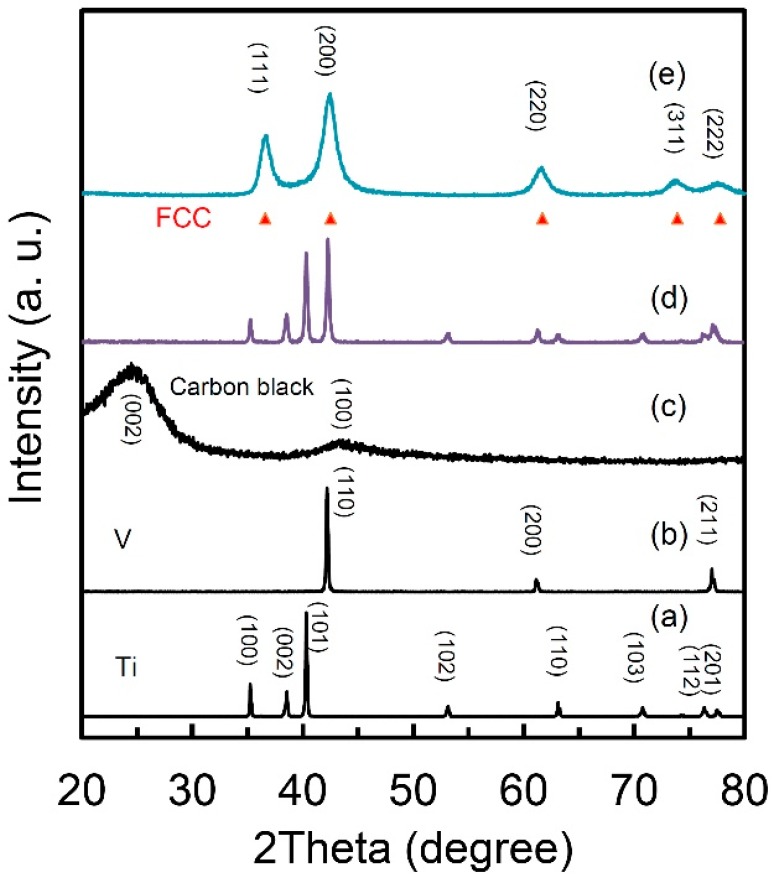
X-ray diffraction pattern of (**a**) Ti; (**b**) V; (**c**) carbon black; (**d**) mixture of Ti_50_V_50_+10 wt.% carbon black and (**e**) Ti_50_V_50_-10% C alloy with face-centered cubic (FCC) structure after 10 h mechanical alloying process.

Figure 1 presents the X-ray diffraction (XRD) curves of the starting materials of Ti (a), V (b) and carbon black (c), the mixture before milling and the obtained Ti_50_V_50_-10% C alloy with a FCC lattice structure. The intensities of the diffraction peaks were normalized to clearly present the phase compositions of each sample. The Ti, V and carbon black samples are with only pure Ti, V and C phases, respectively. The two peaks at around 24° and 44° in Figure 1c are reflections (002) and (100) of graphite indicating graphitization of carbon. After the 10 h milling, there is only one set of diffraction peaks in perfect accordance with a FCC lattice (lattice parameter a= 4.2712 Å, face-cubic centered, space group: *Fm-3m* No. 225, theoretic density: 6.65 g·cm^−3^, experimental density: 5.1 g·cm^−3^). The obvious peak broadening indicates a nanocrystalline microstructure in the sample and the grain size is calculated to be 60 nm. Our experimental density result of 5.1 g cm^−3^ (theoretic density: 6.65 g·cm^−3^) provides strong evidence to large volume of dead pores and/or high content of vacancies in this FCC structured Ti-V-based alloy. Nanostructure in hydrogen storage materials usually means large surface area and short diffusion distance for hydrogen atoms, because of which, unique hydrogen storage properties are expected from this alloy. 

**Figure 2:**
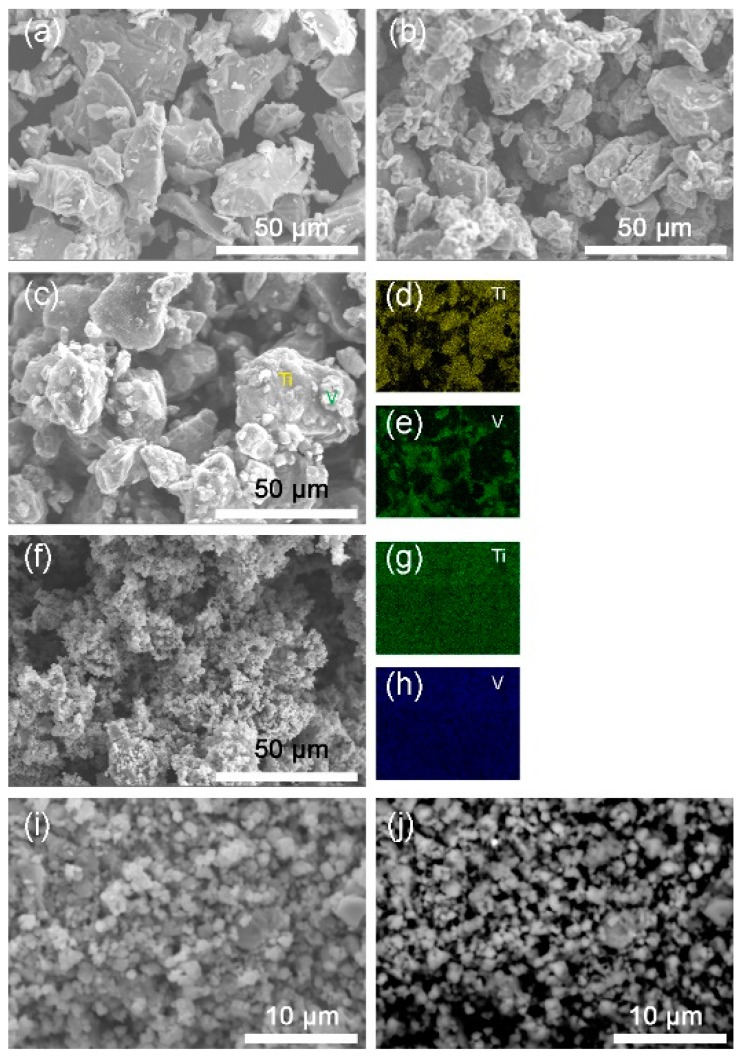
Secondary electron-scanning electron microscope (SE-SEM) images of (**a**) Ti; (**b**) V; (**c**) the mixture of Ti and V; (**f**,**i**) 10 h-milled Ti_50_V_50_-10% C alloy. (**d**,**e**,**g**,**h**) are energy dispersive X-ray spectrometer (EDS) mappings of Ti, V elements for images (**c**,**f**), respectively. (**j**) Backscattered electron-scanning electron microscope (BSE-SEM) image of the milled Ti_50_V_50_-10% C alloy.

Figure 2 shows the SEM images of the Ti, V, the mixture and the 10 h-milled Ti_50_V_50_-10% C alloy with FCC structure. From Figure 2c–e, we may see that the starting sample before milling is just a mixture of Ti and V phases. As seen in Figure 2f–h, after the 10 h mechanical alloying process, Ti and V elements are homogeneously distributed throughout the sample, which means that a uniform compound is formed. From Figure 2i,j, we may see that most of the particles are with a size of 1–3 µm. The morphology of the particles and the grain size in this Ti_50_V_50_−10% C sample is similar to that in our reported Mg-Co-X based alloys [23,28,29], which may absorb hydrogen at −15 °C—the lowest temperature reported to date for solid state materials, as far as we know. We may expect good hydrogen absorption properties from this as-prepared Ti-V-C alloy and it does show superior hydrogen storage kinetics and reversibility. 

**Figure 3:**
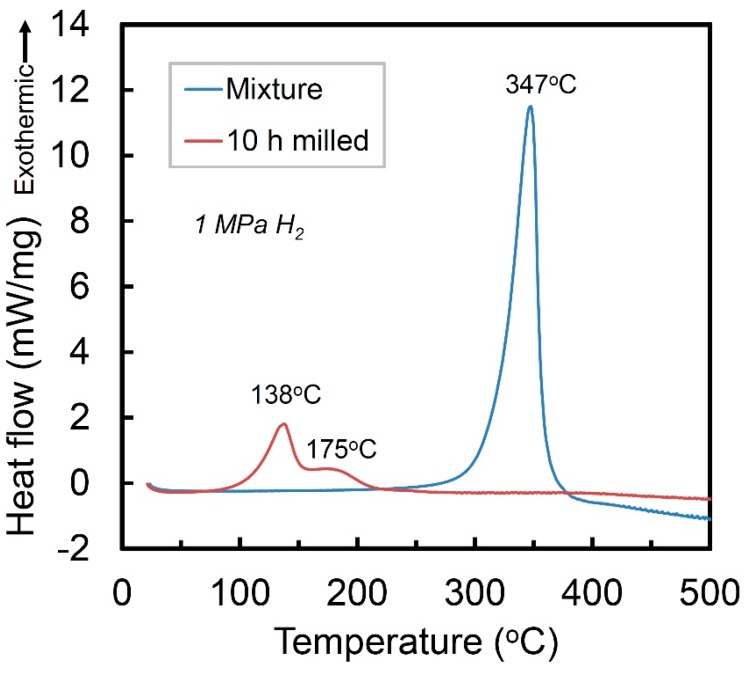
High-pressure DSC curves of the Ti+V+C mixture and the 10 h-milled Ti_50_V_50_-10% C alloy in 1 MPa hydrogen.

Figure 3 presents the high-pressure DSC curves of the mixture of Ti + V + carbon black and the Ti_50_V_50_-10% C alloy with FCC structure, under a hydrogen atmosphere of 1 MPa. The mixture sample shows one exothermic peak at 347 °C, which corresponds to hydrogenation reaction of Ti [22] and the V phase in the mixture cannot absorb hydrogen without activation. The Ti_50_V_50_-10% C alloy with FCC lattice, however, may absorb hydrogen at temperature of 138 °C, which is 209 °C lower than that of the Ti, V and C mixture before mechanical alloying process. It shows a second exothermic stage with a peak at around 175 °C. After the DSC reaction, there is no obvious difference in the XRD curves (will be discussed later), which makes it difficult to clarify the exact mechanisms of the two exothermic peaks. Nevertheless, they are suggested to be hydrogenation reaction of the FCC Ti-V-C alloy, based on the pressure-composition isothermal (PCT) results in the following part. 

**Figure 4:**
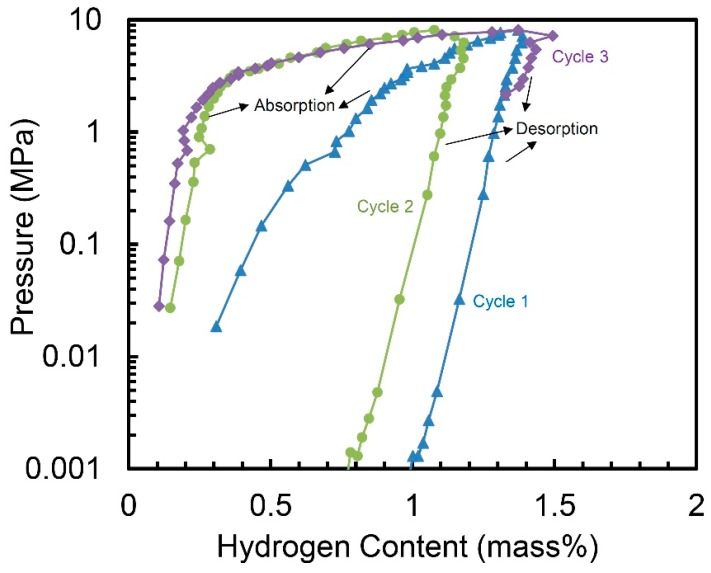
Pressure-composition isotherms (PCT) of the 10 h-milled Ti_50_V_50_-10% C alloy at 30 °C.

Figure 4 demonstrates the hydrogen absorption and desorption properties of the 10 h-milled Ti_50_V_50_-10% C alloy at 30 °C for three cycles, with no activation step. We can see the alloy may absorb hydrogen in the first cycle without any activation and it shows a hydrogen capacity of 1.37 wt.%. The hydrogen storage capacity of the second cycle is a little smaller—1.18 wt.%. But for the third cycle, the capacity goes up to 1.49 wt.%, which is even higher than the one in the first cycle. Although the desorption process of the three cycles in Figure 4 does not clearly show the total release of the hydrogen. However, because the hydrogen absorption capacity in the second and third cycle is similar with the one in the first cycle, we believe the hydrogen can be completely released out during the evacuation process after each desorption cycle. 

Although hydrogen storage capacity of 1.5 wt.% is not a very exciting number, this work is the first case to report Ti-V based alloy with a FCC lattice structure showing reversible hydrogen storage ability without activation. The difficult activation process of Ti-V based hydrogen storage alloys with BCC structure is one of the greatest disadvantages [7]. The FCC lattice structure and fine nanocrystalline with a grain size of 60 nm in this alloy are thought to be the beneficial factors. 

**Figure 5:**
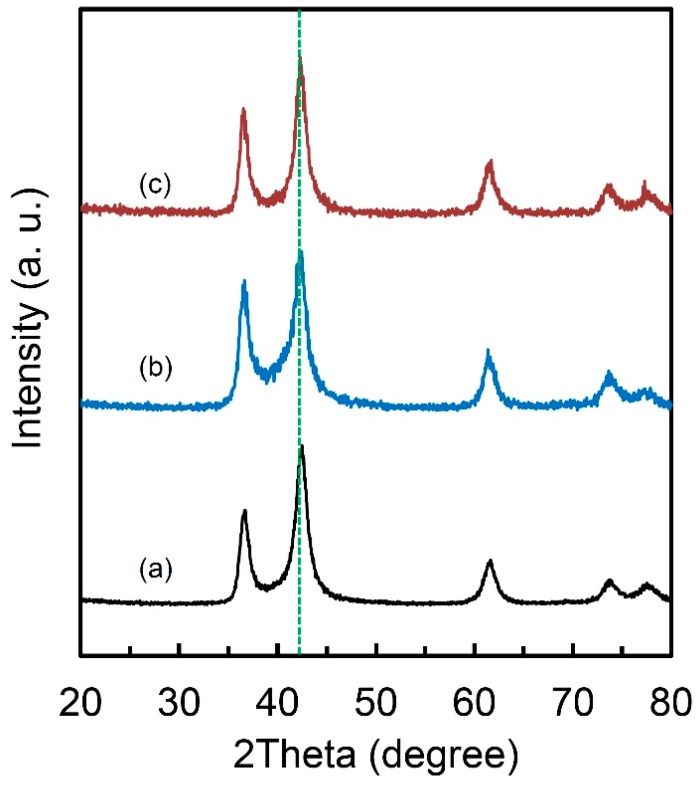
X-ray diffraction curves of (**a**) Ti_50_V_50_-10 wt.% C alloy; (**b**) Ti_50_V_50_-10 wt.% C alloy after PCT measurement and (**c**) Ti_50_V_50_-10 wt.% C alloy after DSC measurement in 1 MPa hydrogen.

It is noteworthy that this special FCC lattice structure could maintain even after PCT and DSC treatments (Figure 5). Which means that this lattice keeps the original structure and does not present apparent lattice expansion during hydrogen absorption/desorption and it is beneficial to the good cycle performance for hydrogen storage. This unique characteristic also makes the packing of the materials much easier when building a hydrogen storage system using this kind of materials. From the XRD result (Figure 1e), the structure of our obtained Ti-V-C alloys are similar with Ti and V carbides. However, because of small neutron scattering length of V, it is difficult to determine the definite local structure even the neutron diffraction technology was applied. XRD also could not tell Ti from V due to the similar structure factors. In addition, the Ti and V carbides normally could not absorb hydrogen unless there exists so-called long-range ordered carbon vacancies and dislocations [30,31,32,33,34]. Our obtained Ti-V-C alloys, which are very likely to possess much carbon vacancies and dislocations based on the smaller experimental density (5.1 g·cm^-3^) compared to the theoretical one, shows unique hydrogen storage properties. For this lattice structure, Ti, V and C atoms are with a simple cubic packing if we consider Ti, V and C as the same atom, with a packing efficiency of only 52%. This packing efficiency is much lower than other FCC (74%), HCP (74%) or even BCC (68%) structure, which possibly implies a high diffusion kinetics of hydrogen atoms in the alloys based on the factor that hydrogen diffusion rate in BCC lattice with a lower packing efficiency than FCC structure is several orders of magnitude higher than in FCC lattice [35]. It remains challenge to figure out the local structure of the as-prepared Ti-V-C alloys, but the combined techniques of XRD and neutron scattering should be taken into consideration, which gives a new direction to develop novel hydrogen storage materials. It is rational to think that the metastable nanophase fabricated by ball milling is the major factor for the good hydrogen storage kinetics [15,29,36]. Particles after milling usually have many cracks, new surface area and small size, indicating that they have more hydrogen transfer paths for hydrogen diffusion in absorption and desorption processes [37]. It is also reported that mechanical milling combined with the catalysts not only improves the hydrogenation kinetics but changes the reaction mechanism from surface penetration to diffusion [38]. However, the intrinsic kinetics mechanism of this Ti-V-C sample needs further study and model building to further understand the mechanism itself; this is under study. Moreover, the lower packing efficiency and the microstructure characteristics of homogeneous particle size of a few micrometers and grain size of 60 nm in the alloy, which normally is not achievable in Ti-V alloys by ball-milling method, are thought to the beneficial factors of good hydrogen storage kinetics in the Ti-V-C alloys.

## 3. Materials and Methods

Ti_50_V_50_ alloys were synthesized from Ti and V metals (325-mesh, purity > 99.5%) by mechanical alloying method. Carbon black was added during the milling process. Without carbon black, the sample would stick to the walls of the milling vessel and to the balls. For a typical milling process, 0.5 g mixture of Ti and V powder with an atomic ratio of 50:50 and 0.05 g (10 wt.%) carbon black were put into a 45 mL stainless steel vessel. Ten stainless steel milling balls with a diameter of 0.7 cm and an average weight of 1.5 g were used. The ball to sample ratio is 30:1.

The X-ray diffraction (XRD) measurements were carried out using a Rigaku diffractometer (Ultima IV) (Tokyo, Japan) with Cu K*α* radiation at a generator voltage of 40 kV and a current of 40 mA, to obtain the phase information of the samples. The grain size was calculated based on the Scherrer equation: D = Kλ/(B cosα), D is the grain size, K is the Scherrer constant, λ is the wavelength of X-ray, B is the FWHM, α is Bragg diffraction angle. The analysis of the microstructure and elemental information was conducted using scanning electron microscope (SEM) (S3400N, Hitachi) (Tokyo, Japan). It investigates samples by monitoring secondary electron (SE) and backscattered electron (BSE) signals. The SEM apparatus has an energy dispersive X-ray spectrometer (EDS) (Tokyo, Japan) attachment. Differential scanning calorimetry (DSC) measurements under hydrogen atmosphere were carried out to study the hydrogen absorption ability and thermodynamics properties of the alloys, through a Rigaku TP-8230 HP apparatus (Tokyo, Japan) under a constant hydrogen pressure of 1 MPa with a flow rate of 200 mL/min. The pressure-composition isothermal (PCT) curves were measured at an automatic Sievert-type apparatus from JMC Corporation (Kanagawa, Japan). The measurements were conducted under a hydrogen pressure up to 8 MPa, at a temperature of 30 °C. Pressure change smaller than 0.00001 MPa in 10 min will be taken as equilibrium.

## 4. Conclusions

In summary, for the first time, the Ti_50_V_50_-10% C alloy with a FCC lattice and a nanocrystalline microstructure was synthesized by mechanical alloying method and the alloy may show a reversible hydrogen storage capacity of ca. 1.5 wt.% at 30 °C, without any activation process. The good kinetics is attributed to the FCC lattice with low metal packing efficiency, micrometer scale particles and nanocrystalline structures of the alloy. Moreover, how to figure out the definite local structure needs further investigation and it will be helpful for us to have a deep comprehension to the special properties. Overall, the results reported here may indicate a new research direction of the development of novel hydrogen storage materials.

## References

[1] Schlapbach L., Zuttel A. (2001). Hydrogen-storage materials for mobile applications. Nature.

[2] Buckley C.E., Chen P., van Hassel B.A., Hirscher M. (2016). Hydrogen-based Energy Storage (IEA-HIA Task 32). Appl. Phys. A.

[3] Mohtadi R., Orimo S.-I. (2016). The renaissance of hydrides as energy materials. Nat. Rev. Mater..

[4] Wang H., Lin H.J., Cai W.T., Ouyang L.Z., Zhu M. (2016). Tuning kinetics and thermodynamics of hydrogen storage in light metal element based systems—A review of recent progress. J. Alloys Compd..

[5] Yang J., Sudik A., Wolverton C., Siegel D.J. (2010). High capacity hydrogen storage materials: Attributes for automotive applications and techniques for materials discovery. Chem. Soc. Rev..

[6] Shao H., He L., Lin H., Li H.W. (2018). Progress and Trends in Magnesium-Based Materials for Energy-Storage Research: A Review. Energy Technol..

[7] Ono S., Nomura K., Ikeda Y. (1980). The Reaction of Hydrogen with Alloys of Vanadium and Titanium. J. Less-Common Met..

[8] Matsuda J., Akiba E. (2013). Lattice defects in V-Ti BCC alloys before and after hydrogenation. J. Alloys Compd..

[9] Kim H., Sakaki K., Nakamura Y. (2014). Improving the Cyclic Stability of V-Ti-Mn BCC Alloys Using Interstitial Elements. Mater. Trans..

[10] Suwarno S., Solberg J.K., Krogh B., Raaen S., Yartys V.A. (2016). High temperature hydrogenation of Ti-V alloys: The effect of cycling and carbon monoxide on the bulk and surface properties. Int. J. Hydrogen Energy.

[11] Banerjee S., Kumar A., Ruz P., Sengupta P. (2016). Influence of Laves phase on microstructure and hydrogen storage properties of Ti-Cr-V based alloy. Int. J. Hydrogen Energy.

[12] Zhu J.B., Ma L.Q., Liang F., Wang L.M. (2015). Effect of Sc substitution on hydrogen storage properties of Ti-V-Cr-Mn alloys. Int. J. Hydrogen Energy.

[13] Qiu S.J., Huang J.L., Chu H.L., Zou Y.J., Xiang C.L., Zhang H.Z., Xu F., Sun L.X., Zhou H.Y. (2015). Influence of boron introduction on structure and electrochemical hydrogen storage properties of Ti-V-based alloys. J. Alloys Compd..

[14] Skryabina N., Fruchart D., Shelyapina M.G., Dolukhanyan S., Aleksanyan A. (2013). Phase transformations in Ti–V hydrides. J. Alloys Compd..

[15] Li J.D., Li B., Shao H.Y., Li W., Lin H.J. (2018). Catalysis and Downsizing in Mg-Based Hydrogen Storage Materials. Catalysts.

[16] Gao Q.L., Xia G.L., Yu X.B. (2017). Confined NaAlH_4_ nanoparticles inside CeO_2_ hollow nanotubes towards enhanced hydrogen storage. Nanoscale.

[17] Huang M.H., Ouyang L.Z., Liu J.W., Wang H., Shao H.Y., Zhu M. (2017). Enhanced hydrogen generation by hydrolysis of Mg doped with flower-like MoS_2_ for fuel cell applications. J. Power Sources.

[18] Lin J., Cao Z.Y., Sun L.S., Liang F., Wang L.M. (2017). Improved electrochemical performance of Ti_1.4_V_0.6_Ni hydrogen storage alloy in its composite with LiAlH_4_. J. Alloys Compd..

[19] Yang T., Liang C.Y., Wang X.H., Wang H.S., Yuan Z.M., Yin F.X., Li Q., Zhang Y.H. (2017). Effect of graphite (GR) content on microstructure and hydrogen storage properties of nanocrystalline Mg_24_Y_3_-Ni-GR composites. J. Alloys Compd..

[20] Li B., Li J., Shao H., He L. (2018). Mg-Based Hydrogen Absorbing Materials for Thermal Energy Storage—A Review. Appl. Sci..

[21] Shao H., Asano K., Enoki H., Akiba E. (2009). Fabrication and hydrogen storage property study of nanostructured Mg-Ni-B ternary alloys. J. Alloys Compd..

[22] Shao H., Felderhoff M., Schuth F. (2011). Hydrogen storage properties of nanostructured MgH_2_/TiH_2_ composite prepared by ball milling under high hydrogen pressure. Int. J. Hydrogen Energy.

[23] Shao H.Y., Asano K., Enoki H., Akiba E. (2009). Preparation and hydrogen storage properties of nanostructured Mg-Ni BCC alloys. J. Alloys Compd..

[24] Shao H.Y., Matsuda J., Li H.W., Akiba E., Jain A., Ichikawa T., Kojima Y. (2013). Phase and morphology evolution study of ball milled Mg-Co hydrogen storage alloys. Int. J. Hydrogen Energy.

[25] Li J., Xu J., Li B., He L., Lin H., Li H.-W., Shao H. (2018). Advanced sem and tem techniques applied in Mg-based hydrogen storage research. Scanning.

[26] Li B., Li J., Shao H., Li W., Lin H. (2018). Synthesis, Morphology, and Hydrogen Absorption Properties of TiVMn and TiCrMn Nanoalloys with a FCC Structure. Scanning.

[27] Shao H., Asano K., Enoki H., Akiba E. (2008). Correlation study between hydrogen absorption property and lattice structure of Mg-based BCC alloys. Int. J. Hydrogen Energy.

[28] Shao H., Asano K., Enoki H., Akiba E. (2009). Fabrication, hydrogen storage properties and mechanistic study of nanostructured Mg_50_Co_50_ body-centered cubic alloy. Scr. Mater..

[29] Shao H., Xin G., Zheng J., Li X., Akiba E. (2012). Nanotechnology in Mg-based materials for hydrogen storage. Nano Energy.

[30] Gringoz A., Glandut N., Valette S. (2009). Electrochemical hydrogen storage in TiC_0. 6_, not in TiC_0.9_. Electrochem. Commun..

[31] Ding H., Fan X., Li C., Liu X., Jiang D., Wang C. (2013). First-principles study of hydrogen storage in non-stoichiometric TiC_x_. J. Alloys Compd..

[32] Nguyen J., Glandut N., Jaoul C., Lefort P. (2015). Hydrogen insertion in substoichiometric titanium carbide. Int. J. Hydrogen Energy.

[33] Nozaki T., Homma H., Hatano Y. (2011). Pressure-Composition Isotherms of TiC_1−x_–H System at Elevated Temperatures. Mater. Trans..

[34] Takahashi J., Kawakami K., Tarui T. (2012). Direct observation of hydrogen-trapping sites in vanadium carbide precipitation steel by atom probe tomography. Scr. Mater..

[35] Völkl J., Alefeld G., Nowick A., Burton J. (1975). Diffusion in Solids.

[36] Luo Q., Li J., Li B., Liu B., Shao H., Li Q. (2019). Kinetics in Mg-based hydrogen storage materials: Enhancement and mechanism. J. Magnes. Alloys.

[37] Song M.Y. (2003). Hydriding kinetics of a mechanically alloyed mixture Mg–10wt% Ni. Int. J. Hydrogen Energy.

[38] Liu J., Zhang X., Li Q., Chou K.-C., Xu K.-D. (2009). Investigation on kinetics mechanism of hydrogen absorption in the La_2_Mg_17_-based composites. Int. J. Hydrogen Energy.

